# Prevalence of Atrial Fibrillation and Intervention Therapy in Cardiac Amyloidosis

**DOI:** 10.31083/RCM37064

**Published:** 2025-05-22

**Authors:** Runzhe Wu, Xiaojun Xia, Yongquan Niu, Feiyu Chen, Jia Zhu, Haodong Jiang, Congying Wang, Yunpeng Jin

**Affiliations:** ^1^Department of Cardiology, The Fourth Affiliated Hospital of School of Medicine, and International School of Medicine, International Institutes of Medicine, Zhejiang University, 322000 Yiwu, Zhejiang, China

**Keywords:** cardiac amyloidosis, arrhythmias, atrial fibrillation, therapy, catheter ablation

## Abstract

Atrial fibrillation (AF) is a common and serious arrhythmia that frequently complicates cardiac amyloidosis (CA), a rare condition characterized by amyloid deposits in the heart. The coexistence of AF in CA patients significantly increases the risk of heart failure, stroke, and other life-threatening complications; however, the therapeutic approach to managing AF in CA patients remains underexplored. Thus, this review discusses the features of AF in CA patients, recent research on the development of effective treatment options, and strategies for future therapies. A comprehensive review of the literature was conducted, assessing the epidemiology of AF in CA, the challenges in treatment, and the available intervention strategies, with a particular emphasis on catheter ablation and anticoagulation therapy. AF is highly prevalent in CA patients, with incidence rates reaching 88%. The presence of amyloid deposits exacerbates the risk of arrhythmias, leading to increased morbidity and mortality. Traditional risk stratification models, such as the Congestive Heart Failure, Hypertension, Age ≥75 [Doubled], Diabetes Mellitus, Prior Stroke or Transient Ischemic Attack [Doubled], Vascular Disease, Age 65–74, Female (CHA_2_DS_2_-VASc) score, have limited effectiveness in CA patients. Anticoagulation therapy, particularly direct oral anticoagulants, is recommended to prevent thromboembolic events, though individualized risk assessment is crucial. Catheter ablation has shown promise in improving outcomes, including reducing hospitalization rates and mortality. However, the benefits of catheter ablation remain controversial in light of recent studies suggesting potential risks such as prolonged hospital stays and higher economic burdens. AF is a significant and often fatal complication of CA. The CHA_2_DS_2_-VASc score has limitations in assessing thrombotic risk in CA patients; meanwhile, speckle-tracking echocardiography (STE) has been shown to indirectly predict the danger of thrombosis in these patients. Therefore, the effect of conducting STE on CA patients needs to be further validated. While current therapies, including anticoagulation and catheter ablation, offer some benefits, their effectiveness remains uncertain due to the complexity of the pathophysiology of CA and limited high-quality studies. Future research should focus on developing amyloid-targeted therapies and conducting randomized trials to optimize AF management in CA patients to improve survival and quality of life.

## 1. Introduction

Atrial fibrillation (AF) is one of the most prevalent cardiac arrhythmias, associated with both multiple non-modifiable factors (such as age, sex, genetics, and race) and modifiable risk factors including smoking, usage of alcohol, sleep apnea, obesity, hypertension, diabetes, and lifestyle. Various cardiomyopathies contribute to the structural abnormalities in the heart that serve as substrates for the development of AF [1]. Cardiac amyloidosis (CA) results in damage in the mechanical structure of the left atrium as well as pressure overload, contributing to electrophysiologic changes, which predisposes patients to AF [2]. CA patients with AF have an increased risk of thrombosis, heart failure, sudden cardiac arrest and death [3]. Despite the increased potential hazards and high prevalence, there is limited knowledge regarding the outcomes of AF ablation and other interventions in patients with CA. Furthermore, medical therapy remains uncertain due to the limitations of the Congestive Heart Failure, Hypertension, Age ≥75 [Doubled], Diabetes Mellitus, Prior Stroke or Transient Ischemic Attack [Doubled], Vascular Disease, Age 65–74, Female (CHA_2_DS_2_-VASc) score. Therefore, we performed this review to determine the potential role of catheter ablation and other therapeutic interventions in CA patients with AF.

## 2. Amyloidosis and Cardiac Involvement

**Fig. 1.**
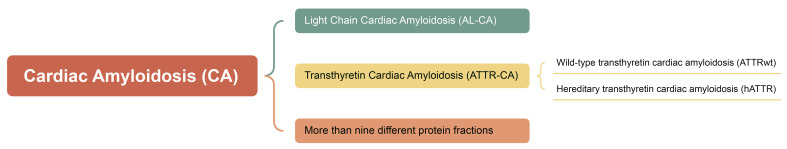
**The typical categories of myocardial amyloidosis**.

Amyloidosis is a collection of rare diseases caused by abnormal folding or aggregation of proteins transforming into amyloid fibers, which are deposited in extracellular tissues and contribute to abnormal cell function resulting in damage to multiple organs. The disease is commonly classified as systemic or localized and further classified in accordance with the site of precursor protein production and amyloid deposition [2, 4, 5]. There are at least 30 types of precursor proteins that have a propensity to form amyloid fibrils [5, 6, 7], and more than nine different protein fractions are considered to cause CA [8]. Among them are 2 common amyloid proteins capable of infiltrating the myocardium: monoclonal immunoglobulin light chain and transthyretin [7]. The typical categories of myocardial amyloidosis are displayed in Fig. 1.

Light chain (AL) amyloidosis is a rare disease caused by over-proliferation of monoclonal plasma cells that produce immunoglobulin κ or λ light chains that are abnormally β-folded and unable to be cleared, some of which are associated with hematologic malignancies including multiple myeloma (MM), Waldenström macroglobulinemia (WM) and non-Hodgkin’s lymphoma. Approximately 10% of amyloidosis patients have a comorbid MM [4, 9, 10, 11]. The existing study shows little geographic variation in the incidence of AL but there are large regional disparities in the rates of diagnosis [12]. From 1998–2018, approximately 74,000 cases of monoclonal immunoglobulin light chain amyloidosis were diagnosed worldwide. Globally, the prevalence rate per million people is about 51.27, with the lowest in Brazil (32.22/million) and reaching 71.08/million in Japan [11]. The median time from the onset of symptoms to the diagnosis of AL is 2.7 years, with 50% of patients visiting 5 or more physicians before the diagnosis is finally made. AL causes organ hypoplasia, multiple organ damage, and even organ failure, and about 24%–37% of patients die within 6 months of diagnosis [13, 14].

The transthyretin (TTR), known as prealbumin, is a tetrameric transport protein originally produced in the liver and consisting of 127 amino acids at 56 kDa. Following the exposure to certain factors, such as aging or genetic mutations, TTR tetrameric proteins are deconstructed into monomers and incorrectly aggregated into amyloid. Two types of transthyretin cardiac amyloidosis (ATTR-CA) are present, an aging-associated amyloidosis named wild-type transthyretin cardiac amyloidosis (ATTRwt) and a heritable type associated with a point mutation in the *TTR gene* at 110 known loci, hereditary transthyretin cardiac amyloidosis (hATTR) [2, 6, 8, 15, 16]. There are no precise statistics on the incidence and prevalence of ATTR-CA [16, 17, 18, 19]. The mean age of onset of ATTRwt is 75 years and the median survival after diagnosis without therapeutic intervention is only 3.5 years. The age of onset, presenting symptoms (cardiac, neurologic, or mixed lesions), and progression of hATTR are largely dependent on the type of mutation. hATTR can occur from 30 to 80 years of age, with a median survival of only 2.5 years for those who are diagnosed but refuse treatment [20]. ATTR-CA has no significant differences in gender, clinical phenotype, disease progression, and prognosis [21].

CA is a cardiomyopathy characterized by extracellular amyloid deposits throughout the heart. Deposits of amyloid can thicken the ventricular wall, leading to centripetal ventricular remodeling, resulting in ejection fraction preserved heart failure (HFpEF), as well as atrial enlargement due to increased pressure gradients. When amyloidosis involves the cardiac conduction system, it can often lead to arrhythmias such as atrial flutter, atrial fibrillation, atrial tachycardia, and atrioventricular block [2, 15, 22, 23]. Based on the significant impact of CA on the cardiovascular system, myocardial involvement is the primary morbid and lethal factor in systemic amyloidosis [22, 24].

## 3. Atrial Fibrillation Associated With Myocardial Amyloidosis

Arrhythmias are a common complication in patients with CA. The incidence of arrhythmias in patients with CA ranges from 10% to 88%, and includes atrial flutter, atrial fibrillation, atrioventricular block, and ventricular arrhythmias. It is not uncommon for severe myocardial amyloidosis-associated arrhythmias to result in cardiac arrest or sudden cardiac death. The incidence is closely related to the severity of the disease, electrophysiologic abnormalities, and the amount of amyloid deposition [25, 26, 27, 28, 29, 30].

Atrial flutter or fibrillation is the most common type of arrhythmia in patients with CA, and approximately 40% of patients with CA have comorbid AF [31, 32]. A multicenter study by Guilio Sinigiani *et al*. [33] showed that the presence of any degree of intra-atrial block (IAB) significantly increased the risk of atrial fibrillation in patients with CA by approximately 2.2-fold (*p* = 0.041).

### 3.1 Prevalence of Atrial Fibrillation in CA

The prevalence of atrial arrhythmias, i.e., atrial flutter (AFL) and AF, has been reported in the literature to be as high as 88%, which is the most significant contributor to the disease burden of CA. Although most studies do not report the exact incidence of atrial flutter, evidence suggests that the incidence of atrial flutter in arrhythmias associated with CA is much smaller than that of AF [2, 25, 26, 27, 28, 31, 34, 35, 36, 37, 38, 39, 40]. Atrial arrhythmias are common in patients with familial amyloidosis, particularly AF, which has a prevalence of more than 40% and significantly increases the risk of thromboembolism [26]. In patients with isolated atrial amyloidosis, there is a significant increase in atrial arrhythmias (e.g., AF) with a prevalence of more than 30%–50% [27]. In a study from the Mayo Clinic, amyloid was detected in 173 (45%) of 383 tissue specimens from 345 patients who underwent resection of the atrial appendage in 160 (46%) patients, and 271 (78.6%) patients had been diagnosed with atrial fibrillation prior to surgery [28]. The prevalence of AF in patients with CA exceeded 40% in several large, global, multicenter studies in which the type of myocardial amyloid has been defined [33, 41]. A study by Syed Bukhari *et al*. [42] exhibited an extremely high prevalence of AF in ATTRwt patients, reaching as high as 88%. These results indicate that the prevalence of AF in patients with CA is much higher than the prevalence in the general population.

Between 30% and 70% of patients diagnosed with ATTR-CA have concomitant atrial flutter or AF. In a study of 345 patients, there was a significant correlation between atrial amyloid deposition and the development of AF, with a prevalence close to 60% [28]. An observational study from Cedars-Sinai Medical Center comparing ATTR-CA with light chain cardiac amyloidosis (AL-CA) found that patients with AF with ATTR-CA were more likely to have a stroke and had a higher rate of anticoagulant therapy and left appendage closure, suggesting that ATTR-CA is more prone to the risk of thrombosis. There were however no strokes in these patients with ATTR-CA and AF who underwent left appendage closure during the follow-up period [40]. Many studies have revealed that patients with myocardial amyloidosis combined with AF have increased atrial enlargement and a significant severe decrease in left ventricular ejection fraction (LVEF), resulting in increased incidence of hospitalization for heart failure [33, 40, 41, 43, 44, 45, 46].

### 3.2 Thrombosis Risk in CA Patients With AF

#### 3.2.1 Limitations of the CHA_2_DS_2_-VASc Score 

Although it is well known that CA is associated with an elevated risk of thrombosis, comorbid AF further increases the risk of intracardiac thrombosis and stroke. Unfortunately, CHA_2_DS_2_-VASc scores appear to have limited ability to predict the risk of thrombotic events in these patients [2, 34, 40, 43, 44, 45, 46]. In a large multicenter longitudinal study in the United States that enrolled approximately 1200 patients with CA of various pathology types, 13.6% of patients had already had an embolic event (including stroke, transient ischemic attacks, and peripheral embolism) prior to participation in the study, and over a median observation time period of 19.9 months (interquartile range, IQR 9.9–35.5), 3.44% of the patients had an embolic event, with more than half of them having AF at the time of recruitment into the study, for an overall prevalence of 16.2% [29]. In patients with or without AF, the risk of embolism increased in descending order from those in sinus rhythm who were not taking oral anticoagulants to those with AF taking oral anticoagulants to those with AF who were not taking oral anticoagulants (1.3, 1.7, and 4.8 per 100 patients), respectively. Another retrospective cohort study that included 382 patients with amyloidosis showed similar results, with the incidence of cerebrovascular accidents being approximately 16% and showed a significant incidence of cerebrovascular accidents in patients with concomitant AF compared with patients without AF (20% vs. 9%, *p* = 0.005) [43]. Nevertheless, in the multicenter real-world cohort by Francesco Cappelli *et al*. [46], only 7.6% of patients experienced an arterial thromboembolic event over a median observation time of 19 months, of which more than 90% were cerebrovascular events, with arterial thromboembolism as the first manifestation of myocardial amyloidosis in close to one-third of the patients, and recurrent embolic events occurring in close to 20% of the patients. No more than half of the patients with arterial thrombo-embolic events (AEs) in AL and mutant ATTR-CA had a CHA_2_DS_2_-VASc score of more than 3.

#### 3.2.2 Predicting Thrombosis Risk

Left atrial (LA) and left atrial appendage (LAA) dysfunction is considered to be the predominant mechanism of thrombosis formation in non-valvular atrial fibrillation (NVAF) [47]. Transthoracic echocardiography (TTE) implemented with speckle tracking echocardiography (STE) analysis plays a role in defining the abnormal mechanisms, which improves the assessment of thrombotic risk in NVAF patients. A previous study found that peak negative strain rate, associated with both maximum emptying velocity and maximum filling velocity in the LAA, along with time-to-peak positive strain, were independent predictors of LAA thrombosis (LAAT) [47]. In another in-depth study, more factors, such as LVEF, average E/e’ ratio and LA peak positive global atrial strain (LA-GSA+), were identified as independent contributors of LAAT [48]. However, these studies demonstrated a lack of evidence in CA patients.

### 3.3 Prognostic Predictions Factors

#### 3.3.1 N-terminal Pro-B-type Natriuretic Peptide (NT-proBNP)

Recently, several researchers have proposed NT-proBNP as one of the independent prognostic factors of systemic amyloidosis involvement in the heart, as well as for monitoring changes in the disease and predicting the prognosis of these patients. High levels of NT-proBNP are generally associated with malignant arrhythmias, such as ventricular arrhythmias and persistent AF, and decreases in the level of NT-proBNP are usually associated with an improvement in prognosis. In a single-center case-control study, Giovanni Palladini *et al*. [49] suggested that NT-proBNP, with a sensitivity of 100% for assessing myocardial involvement in systemic amyloidosis, is a significant marker of cardiotoxicity due to amyloid light chains, the most sensitive indicator of myocardial dysfunction and the most pivotal prognostic determinant of AL-CA. NT-proBNP is used in CA, not only to assess the degree of cardiac involvement, but also to predict the risk of malignant arrhythmias. High levels of NT-proBNP (>5000 pg/mL) are usually associated with malignant arrhythmias such as ventricular arrhythmias and AF. The use of pharmacologic therapy and implantable cardioverter defibrillator (ICD) implantation can reduce arrhythmia-related mortality and is particularly effective in patients with high NT-proBNP [50].

#### 3.3.2 Myocardial Strain Parameters

STE represents a novel imaging technique that holds promise for the early detection of subclinical myocardial dysfunction, characterized by a reduction in left ventricular (LV) global longitudinal strain (GLS), despite the preservation of LVEF. Using 2-dimensional strain echocardiography, researchers from Emory University and the Cleveland Clinic Foundation revealed that CA patients have significantly worse dysfunction in global myocardial deformation compared to those with either hypertrophic cardiomyopathy or hypertension [51]. Further study by Sebatian J. Buss *et al*. [52] confirmed that NT-proBNP demonstrated a significant correlation with longitudinal strain (LS) and two-dimensional (2D)-GLS in patients with AL. Thus, LS and GLS are prognostic indicators in CA patients.

## 4. Advances in the Therapy of CA-Associated Atrial Fibrillation

### 4.1 Medication Treatment

Given the increased thrombo-embolic risk in CA patients with AF and to limited prognostic role of the CHA_2_DS_2_-VASc system, the European Society of Cardiology (ESC) has recommended oral anticoagulation in their guideline for the management of cardiomyopathies as class I level of evidence B regardless of the CHA_2_DS_2_-VASc score, although there are no randomized controlled trials (RCTs) and only retrospective evidence was provided [53]. Direct oral anticoagulation (DOCA) is the recommended medication for preventing embolism in CA-associated AF, but it needs to be applied after careful assessment of the risk of bleeding. There are no definitive studies on the choice of anticoagulation. Heparin products, vitamin K antagonists, and DOCA are widely used, and the latter two are more commonly used in clinical practice. Mitrani *et al*. [54] found no significant difference in thromboembolic events and risk of major bleeding between vitamin K antagonists (e.g., warfarin), and direct oral anticoagulants, with the rate of thrombotic events during the observation period being 2.9% in patients taking warfarin and 3.9% in patients taking direct oral anticoagulants. Although the risk was higher in the warfarin group after the Hypertension, Abnormal Renal/LiverFunction, Stroke, Bleeding History or Predisposition, Labile INR, Elderly, Drugs/Alcohol Concomitantly (HAS-BLED) score, bleeding events were not significantly increased compared with the direct oral anticoagulant group [54].

### 4.2 Intervention

Catheter ablation has been regarded to the first-line as well as the curative treatment for AF to return to sinus rhythm. However, the superiority of catheter ablation was questioned in recent studies (Table 1) [1, 55, 56, 57, 58, 59, 60]. French researchers, in a study on catheter ablation for patients with CA and atrial arrhythmias, found only modest long-term benefits [55]. Alhassan *et al*. [56] compared catheter ablation therapy between CA patients and those with dilated cardiomyopathy (DCM). The number of patients discharged home following hospitalization and the total hospital charges were not significantly different [56]. Another retrospective observational study was performed with 72 ATTR-CA patients. Ablation for AF was performed in 24 patients with a mean time from diagnosis of ATTR-CA to ablation of 34 months. During the 39 ± 19 months of follow-up, the ablation group achieved significant gains including less hospitalization for heart failure (HF) or arrhythmia (18% for ablation group vs. 72% for non-ablation group, *p* < 0.001), decreased mortality (29% vs. 75%, *p* = 0.01), and improved ejection fraction (EF) (*p* = 0.02) [57]. A recent meta-analysis found that the recurrence rate of AF among CA patients who underwent catheter ablation was 35%, leading to a significant reduction in long-term all-cause mortality [59]. In contrast, a recent review analyzing the National Inpatient Sample (NIS) in the U.S.A found that ablation patients had significantly longer hospital stays, more serious economic burdens and greater probability for acute heart failure [58]. A similar result was found by Eric Black-Maier and his colleagues [60]. The use of catheter ablation resulted in a significant risk of mortality [60].

**Table 1.** **Studies exploring catheter ablation in cardiac amyloidosis with AF**.

Authors	Included period	Median follow-up	Number of included samples	Main outcomes
Philippe Maury *et al*. [55]	2014–2021	19 months	31 CA patients	AA recurrence rate was 45%, including AF 8/14; All-cause mortality was 39%.
			ATTR-CA 61%	
			AL-CA 39%	
Hassan A Alhassan *et al*. [56]	Q4 of 2015–2019		42 CA, 95% ATTR-CA	Proportion of patients discharged home following hospitalization was 97.6%; Total rate of procedural complications was 14.3%.
Alexandros Briasoulis *et al*. [57]	2005–2018	39 ± 26 months	24/72 CA patients with AF underwent ablation	Only 18% hospitalization for HF or arrythmias in the ablation group; 29% mortality rate in the ablation group vs. 75% that of non-ablation group.
Song Peng Ang *et al*. [59]	Up to June 28, 2024		8 studies including 168 CA patients with AA underwent ablation	Total all-cause mortality rate among the ablation group was 0.23; Pooled recurrence of AF was 35%.
Garba Rimamskep Shamaki *et al*. [58]	2016–2021		595/73,160 CA patients with AF underwent ablation from NIS	Significantly more likely to be diagnosed with heart failure (75.54% vs. 65.28%, *p* = 0.042); Significantly longer hospital stays (7 days vs. 5 days, *p * < 0.01).
Eric Black-Maier *et al*. [60]	Jan. 1, 2011 to Dec. 1, 2018.	1 year	10/13 CA patients with AA underwent ablation	Mean time to arrythmia recurrence was 11.9 ± 12.3 months. 1-year mortality rate was 30% and 1-year recurrence rate was also 30%.

The abbreviations in the table: AA, atrial arrhythmias; AF, atrial fibrillation; Q4, the fourth quarter; HF, heart failure; NIS, National Inpatient Sample.

Left atrial appendage closure (LAAC) or left atrial appendage occlusion (LAAO) are traditional interventions to reduce the risk of thrombosis in patients with AF. Recent studies have demonstrated its efficacy in AF with a high risk of bleeding. Ignacio J Amat-Santos *et al*. [61] examined the efficacy of this technique in a population with CA and found that 2-year survival was slightly lower in patients with CA who underwent LAAC compared with the general population but did not show a statistical difference (20% vs. 13.6%). This investigation demonstrated an advantage over patients with myocardial amyloidosis-related AF at two-year follow-up without LAAC intervention in terms of postoperative complications (*p* = 0.248) and showed an improvement over patients with AF associated with CA without LAAC intervention at two-year postoperative follow-up, whereas the rate of postoperative complications was similar to that of the general population (2.5% vs. 2.1%) [61]. In a multicenter European study, there was no significant difference in the prevalence of stroke, major hemorrhage, or peripheral embolism between patients with ATTR-CA AF and AF without this condition within 5 years of LAAC, but there was a statistically significant difference in mortality (40% vs. 19.2%, *p* = 0.001) despite the fact that patients in the ATTR-CA cohort were older, had a higher risk of thrombosis and hemorrhage, had more concomitant diseases, and had poorer left ventricular ejection fraction [61].

## 5. Conclusion and Future Prospects

AF is a prevalent and significant complication of CA, often leading to severe outcomes such as heart failure and stroke. AF is as an independent predictor of mortality in CA patients [57]. The complex pathophysiology, driven by amyloid deposition and myocardial fibrosis, complicates both diagnosis and treatment. While current therapies, including pharmacological agents and interventional therapies, offer inadequate assessment, they underscore the need for treatment regimens that address both the underlying amyloid disease and the arrhythmia itself.

Future researches, focusing on developing amyloid-specific therapies or targeted therapies on CA genes to slow amyloid deposition and reduce arrhythmic burden are necessary. Although the safety and clinical benefits of catheter ablation have been demonstrated in a number of studies, its application remains somewhat controversial because of the lack of relevant evidence of better outcomes. Therefore, more convincing data, such as randomized as well as non-randomized clinical trials aimed at optimizing AF management in CA, will be critical in establishing evidence-based approaches that improve patient outcomes and quality of life.

## Availability of Data and Materials

The original contributions presented in the study are included in the article, further inquiries can be directed to the corresponding author.
